# Validation of the new classification of pauci-immune glomerulonephritis in a United States cohort and its correlation with renal outcome

**DOI:** 10.1186/1471-2369-14-210

**Published:** 2013-10-04

**Authors:** Carla L Ellis, Rebecca L Manno, John P Havill, Lorraine C Racusen, Duvuru Geetha

**Affiliations:** 1 Departments of Pathology, The Johns Hopkins Hospital and School of Medicine, Baltimore, Maryland, USA; 2 Medicine, The Johns Hopkins Hospital and School of Medicine, Baltimore, Maryland, USA; 3 The Johns Hopkins Bayview Medical Center, 301 Mason Lord Drive, Baltimore, MD 21224, USA; 4 Pathology Department, Emory University School of Medicine, Atlanta, GA 30322, USA

**Keywords:** Pauci-immune glomerulonephritis, ANCA vasculitis, Histologic classification, Renal outcome

## Abstract

**Background:**

Renal biopsies provide important diagnostic and prognostic information in ANCA associated glomerulonephritis. A new classification for prognostication of pauci-immune glomerulonephritis (GN) based on four categories (Mixed, Crescentic, Sclerotic and Focal) was proposed by an international working group of renal pathologists (IWGRP). The goal of our study was to apply the proposed classification system to a United States cohort of vasculitis patients and determine the association of IWGRP class with estimated glomerular filtration rate (eGFR) at one year.

**Methods:**

Seventy-six cases of pauci-immune glomerulonephritis diagnosed from 1995 to 2011 from a single center were identified for this retrospective study. Clinical data were collected by abstraction from medical records. Histology was reviewed by a pathologist and classified according to the new classification. MDRD formula was used to calculate eGFR. We correlated IWGRP class to renal function at presentation and at one year. ×^2^, ANOVA, and linear regression analysis were performed as appropriate.

**Results:**

Renal biopsies were categorized as focal: n = 20, crescentic: n = 18, mixed: n = 27, sclerotic: n = 11. The baseline e-GFR was lowest in the crescentic class and highest in the focal class. In linear regression analysis investigating e-GFR at 1 year; age and baseline e-GFR were independent predictors of e-GFR at 1 year.

**Conclusions:**

The e-GFR at diagnosis and age were predictors of e-GFR at 1 year. Pathologic class at diagnosis may also be a helpful tool in risk stratification at diagnosis.

## Background

Pauci-immune crescentic glomerulonephritis accounts for 80% of rapidly progressive glomerulonephritis cases [1-3]. The typical histopathology is a necrotizing glomerulonephritis (GN) with crescents, with a minimal amount of immunoglobulin deposition in vessel walls. The majority of cases of pauci-immune glomerulonephritis can be attributed to a primary systemic small vessel vasculitis such as granulomatosis with polyangiitis (GPA), microscopic polyangiitis (MPA), or eosinophilic granulomatosis with polyangiitis (EGPA, Churg-Strauss syndrome). Renal limited vasculitis (RLV) may also occur [4]. Antineutrophil cytoplasmic antibody (ANCA) is present in 80 to 90% of patients with pauci-immune GN leading to the nomenclature, ANCA associated vasculitis (AAV) [5].

Renal involvement is considered a severe disease manifestation of AAV and progression to end stage renal disease can be prevented by prompt diagnosis and timely initiation of immunosuppressive therapy established by randomized controlled trials [6,7]. Although immunosuppressive therapy may be lifesaving, it can also be associated with short term and long term morbidity and mortality. Therefore, prognostication is important to identify patients with salvageable kidney function and in whom the risks of immunosuppressive therapy are outweighed by its potential benefit. Previous studies have associated the severity of renal dysfunction at diagnosis with reduced renal survival [8,9]. The combination of baseline renal function and renal histology has been shown to be a better predictor of renal outcome than baseline renal function alone [10-18]. Considering the diagnostic and prognostic value of renal histology, a classification system was proposed by an international working group of renal pathologists (IWGRP). The group proposed four categories of glomerular lesions: Focal, Crescentic, Mixed and Sclerotic [19]. The validation cohort for this classification system included 100 patients with AAV from two trials conducted by the European Vasculitis Study Group (EUVAS): CYCAZAREM (CYClophosphamide or AZAthioprine as a remission therapy for vasculitis) [6] and MEPEX (Methylprednisolone versus Plasma Exchange as adjunctive therapy for severe renal vasculitis) [7]. This validation study conducted by EUVAS demonstrated that the different glomerular categories correlated with severity of renal impairment during long term follow up. Importantly, patients in the focal category had the best renal outcomes and those with sclerotic lesions had the worst renal outcomes.

The objective of the study was to validate the classification put forth by the IWGRP in a U.S cohort of patients with pauci-immune GN. Association between histopathologic class and renal function at diagnosis and one year after diagnosis were evaluated.

## Methods

### Study population

The study utilized a retrospective cohort from the Johns Hopkins Hospital, Baltimore, Maryland. Potential patients were identified from the Johns Hopkins Hospital pathology data system via a search between the years of 1995 and 2011 using the term “pauci-immune glomerulonephritis”. “Pauci-immune” was defined as “the intensity of glomerular immunoglobulin staining by direct immunofluorescence assay in renal sections being 0 to 1+, on a_staining scale of 0 to 4 + .” Patients were included if they had renal biopsy specimens with a minimum of 10 glomeruli and a history of ANCA testing. The study protocol was approved by the Johns Hopkins University Institutional Review Board.

Patients were clinically classified as GPA or MPA as defined by the Chapel Hill Consensus Conference [20]. Renal limited vasculitis (RLV) was defined as pauci-immune necrotizing and/or crescentic glomerulonephritis without any overt signs of systemic vasculitis. Patients were followed until end stage renal disease (ESRD), transplant, death, and transfer to another facility with a minimum of 1 year follow-up or until the end of the follow up period which was December 31st 2011.

### Acquisition of clinical data

Patient demographics, clinical features at the time of diagnosis, and timing of renal biopsy (new versus established diagnosis), were abstracted from the clinical records. Need for renal replacement therapy at the time of diagnosis, at one year after diagnosis, and at last follow up were also recorded, along with the details of induction and maintenance immunosuppressive therapy. The outcome of ESRD was defined as need for initiation of renal replacement therapy in the form of dialysis or transplant.

### Laboratory data

Peak serum creatinine at the time of diagnosis and serum creatinine at 6 months, 12 months and 24 months were recorded. Renal function was measured using the 4 variable modification of diet in renal disease (MDRD) formula [21]. The measurement of estimated GFR at 6 months, 12 months and 24 months only included patients without ESRD and not dialysis-dependent. Presence of c-ANCA, p-ANCA, PR3 ELISA and MPO ELISA at the time of diagnosis was recorded. ANCA testing was done by standard indirect immunofluorescence assay on ethanol fixed neutrophils. PR3 and MPO testing was done by direct enzyme linked immunosorbent assay with commercially available kits. ANCA titre of greater than 10 (reference range 0 to 10 units) was considered to be positive for c-ANCA and p-ANCA. PR 3 and MPO levels of greater than 20 units were positive (reference range 1 to 20 units). If the testing for c-ANCA, p-ANCA, PR3 ELISA and MPO ELISA was negative, patients were labeled as having ANCA negative vasculitis.

### Renal histopathology evaluation and classification

Renal biopsies were evaluated by: light microscopy using periodic acid-Schiff, hematoxylin eosin, Masson’s trichrome and methanamine silver stains; direct immunofluorescence for immunoglobulins and complement components and electron microscopy. The following criteria were used to define glomerular abnormalities: “normal” glomeruli with no features of vasculitic lesions, global glomerulosclerosis, intracapillary proliferation, synechiae or focal or segmental glomerulosclerosis; crescentic lesions with purely or partially cellular crescents distinct from fibrous crescents and either segmental or global in morphology: and global glomerulosclerosis when there was more than 80% sclerotic change in the glomerular tuft. The renal histo-pathology was read by 3 different pathologists during this study period. For the purpose of this study, all biopsies were re-analyzed by a single pathologist(CLE) blinded to the clinical data and the cases were categorized based on the results into 4 classes based on the criteria used by the IWGRP: Focal (≥ 50% normal glomeruli), Crescentic (≥ 50% glomeruli with cellular crescents), Mixed (<50% normal, <50% crescentic, <50% globally sclerotic glomeruli) and Sclerotic (≥ 50% globally sclerotic glomeruli) [19]. Tubular atrophy and interstitial fibrosis were scored on a scale of 0 to 3 (0: no abnormalities, 1: <30% affected, 2: 31-60% affected, 3: >60% affected).

### Statistical analyses

Continuous variables were expressed as mean value ± standard deviation. Discrete variables were expressed as proportions. Differences between means and proportions were examined using ANOVA for continuous and Chi-square test for dichotomous variables.

The association between IWGRP class, renal function at presentation (eGFR at time of biopsy), and the outcome of eGFR at one year was assessed by linear regression. Dummy variables were generated based on histopathologic class using the 'focal’ class as the reference group. Kaplan-Meier survival estimates were generated to evaluate time to development of ESRD stratified by renal pathologic class. Statistical analyses were performed using Stata IC 10.0. All reported p-values are two sided with α = 0.05

## Results

### Demographics and clinical characteristics

Seventy six cases of pauci-immune glomerulonephritis from 76 patients were identified. Except for one patient who underwent a repeat renal biopsy, the remaining biopsies represent the first renal biopsy from these patients. Table 1 lists the demographics and baseline characteristics of these patients. The mean age was 58 years. Males comprised 57% of the cohort, and the majority of patients were Caucasian (82%). Sixty two patients (82%) were ANCA positive (c-ANCA: 30, p-ANCA: 32) and the remainder were ANCA negative. Forty three patients (57%) had a phenotype of GPA. The majority of patients in focal (95%), crescentic (89%), and mixed (74%) class had a new diagnosis of vasculitis at the time of biopsy, compared to only 55% of patients in the sclerotic class who had more relapsing disease (p = 0.08) compared to other histologic groups.

**Table 1:** Clinical characteristics of cohort stratified by renal histopathology

	**Crescentic GN n = 18**	**Focal GN n = 20**	**Mixed n = 27**	**Sclerotic n = 11**	**p-value**
Mean age at biopsy, years (±SD)	61 ± 13	56 ± 15	57 ± 17	60 ± 17	0.749
Female (%)	7 (38)	6 (28)	15 (55)	5 (50)	0.354
African-American (%)	1 (5)	5 (24)	7 (26)	1 (10)	0.240
ANCA positive (%)	16 (89)	15 (75)	22 (81)	9 (82)	0.749
cANCA positive (%)	12 (67)	7 (33)	10 (37)	1 (10)	0.018
pANCA positive (%)	4 (22)	8 (40)	12 (44)	8 (73)	0.064
New vasculitis diagnosis at biopsy (%)	16 (89)	18 (90)	20 (74)	6 (55)	0.076
Clinical vasculitis phenotype					
Granulomatosis with polyangiitis (%)	15 (83)	8 (38)	18 (67)	2 (20)	0.002
Microscopic polyangiitis (%)	3 (16)	11 (55)	8 (30)	9 (82)	0.002
Renal limited vasculitis (%)	0 (0)	1 (5)	1 (4)	0 (0)	0.719

### Classification of renal biopsies

Renal biopsies were categorized as focal in 20 patients, crescentic in 18 patients, mixed in 27 patients and sclerotic in 11 patients. The mean number of glomeruli in the biopsies was 29. The mean number of glomeruli in the biopsy sample was significantly higher in the sclerotic class compared to other classes, reflecting parenchymal atrophy with increased density of glomeruli. Patients in the crescentic class were more often c-ANCA positive with GPA phenotype, and patients in the sclerotic class were more often p-ANCA positive with MPA phenotype (p = 0.02 and p = 0.06 for all 4 classes). The proportion of normal glomeruli, glomeruli with cellular crescents, and globally sclerotic glomeruli in each class and the scores for tubular atrophy and interstitial fibrosis is shown in Table 2. These significant differences in distribution of glomerular lesions in the different classes are expected differences confirming class definitions.

**Table 2:** Renal characteristics and outcomes of cohort stratified by renal histopathology

	**Crescentic GN n = 18**	**Focal GN n = 20**	**Mixed n = 27**	**Sclerotic n = 11**	**p-value**
Mean number of glomeruli on biopsy (±SD)	26 ± 11	28 ± 16	26 ± 11	44 ± 32	0.018
Mean % normal glomeruli (±SD)	23 ± 9	70 ± 13	31 ± 14	23 ± 22	<0.001
Mean % cellular crescentic glomeruli (±SD)	63 ± 11	13 ± 12	24 ± 15	14 ± 12	<0.001
Mean % globally sclerotic glomeruli (±SD)	9 ± 8	12 ± 12	20 ± 15	65 ± 10	<0.001
Median interstitial fibrosis score (range)*	1 (0,2)	1 (0,3)	2 (0,3)	2 (2,3)	<0.001
Median tubular atrophy score (range)*	1 (0,2)	1 (0,3)	2 (0,3)	2 (2,3)	<0.001
Mean creatinine (mg/dL) at biopsy (±SD)	5.2 ± 3.2	3.4 ± 4.1	3.8 ± 2.3	3.2 ± 19	0.249
Mean eGFR (mL/min/1.73 m^2^) at biopsy (±SD)	16.4 ± 10.3	50.2 ± 63.4	26.5 ± 23.3	26.5 ± 15.0	0.035
Mean creatinine (mg/dL) at 6 months (±SD)**	2.6 ± 3.1	1.2 ± 0.4	2.1 ± 0.9	2.2 ± 0.7	0.112
Mean eGFR (mL/min/1.73 m^2^) at 6 months (±SD)**	40.8 ± 23.8	76.0 ± 38.7	42.2 ± 29.6	32.8 ± 10.7	0.001
Mean creatinine (mg/dL) at 12 months (±SD)^§^	1.9 ± 0.8	1.2 ± 0.3	2.1 ± 0.8	2.4 ± 1.1	0.001
Mean eGFR (mL/min/1.73 m^2^) at 12 months (±SD)^§^	42.1 ± 22.4	70.8 ± 29.6	37.8 ± 19.8	32.7 ± 15.3	<0.001
Mean creatinine (mg/dL) at 24 months (±SD)^§§^	1.9 ± 0.8	1.1 ± 0.3	1.9 ± 0.7	2.1 ± 0.8	0.007
Mean eGFR (mL/min/1.73 m^2^) at 24 months (±SD) ^§§^	41.7 ± 18.1	76.7 ± 29.5	42.9 ± 21.6	37.8 ± 16.8	0.001
Mean change in eGFR (mL/min/1.73 m^2^) from baseline to 12 mos^§^	25.7 ± 23.4	15.9 ± 47.8	10.4 ± 14.8	1.4 ± 12.3	0.373
Hemodialysis required at presentation (%)	33	20	37	36	0.625
Hemodialysis discontinued with treatment of vasculitis (%)^Δ^	50	75	56	0	0.166
ESRD at 12 months (%)	22	10	19	27	0.549
ESRD at 24 months (%)	22	10	22	36	0.603
Death (%)	17	5	11	0	0.415

*Score 0 = none, 1 = mild, 2 = moderate, 3 = severe.

**data calculated from 60 subjects (n = 3 excluded because of missing data; n = 13 excluded because reached ESRD endpoint within 6 months).

^§^data calculated from 57 subjects (n = 5 excluded because of missing data; n = 14 excluded because reached ESRD endpoint within 12 months).

^§§^data calculated from 44 subjects (n = 16 excluded because of missing data; n = 16 excluded because reached ESRD endpoint within 24 months).

^Δ^percentage calculated from number of individuals in class on hemodialysis at presentation.

### Histologic class and renal function and renal survival

The baseline e-GFR was lowest in the crescentic class and highest in the focal class (16 ml/min/1.73 m^2^ vs 50 ml/min/1.73 m^2^, p = 0.04).Twenty four patients were dialysis dependent at presentation (4 in focal, 4 in sclerotic 6 in crescentic and 10 in mixed class), and 22 of these patients were treated with immunosuppressive drugs. Eleven patients recovered enough renal function to discontinue dialysis (5 in mixed, 3 in focal, 3 in crescentic and none in sclerotic, p = 0.17), and the remaining 13 patients remained dialysis dependent (3 crescentic, 4 sclerotic, 5 mixed and 1 focal). Two patients did not receive any treatment. Both of these patients were dialysis dependent at diagnosis with biopsies classified as sclerotic. Four patients were treated with corticosteroids only (2 in focal and 2 in mixed class) .Initial immunosuppressive regimens included corticosteroids and oral cyclophosphamide (n = 52), corticosteroids and intravenous cyclophosphamide (n = 5), mycophenolate mofetil (n = 5), rituximab (n = 3), azathioprine (n = 1), methotrexate (n = 1), cyclosporine (n = 1), and intravenous immunoglobulin (n = 1). A single patient received steroids, oral cyclophosphamide, and rituximab for initial therapy. None of the patients received plasmapheresis. Among the 13 initially dialysis dependent patients who did not recover, 10 were treated with combination of steroids and cyclophosphamide, 1 received corticosteroids only and 2 did not receive any treatment. Among the 11 initially dialysis dependent patients who recovered, 8 were treated with a combination of steroids and cyclophosphamide, 1 patient was treated with a combination of steroids, rituximab and cyclophosphamide, one patient was treated with steroids and rituximab and a single patient received steroids and mycophenolate mofetil.

Seventy two of seventy four patients treated with immunosuppressive therapy achieved remission of their vasculitis as defined by a Birmingham vasculitis activity score of zero. There were no differences between histologic groups in achievement of remission.

**Figure 1:**
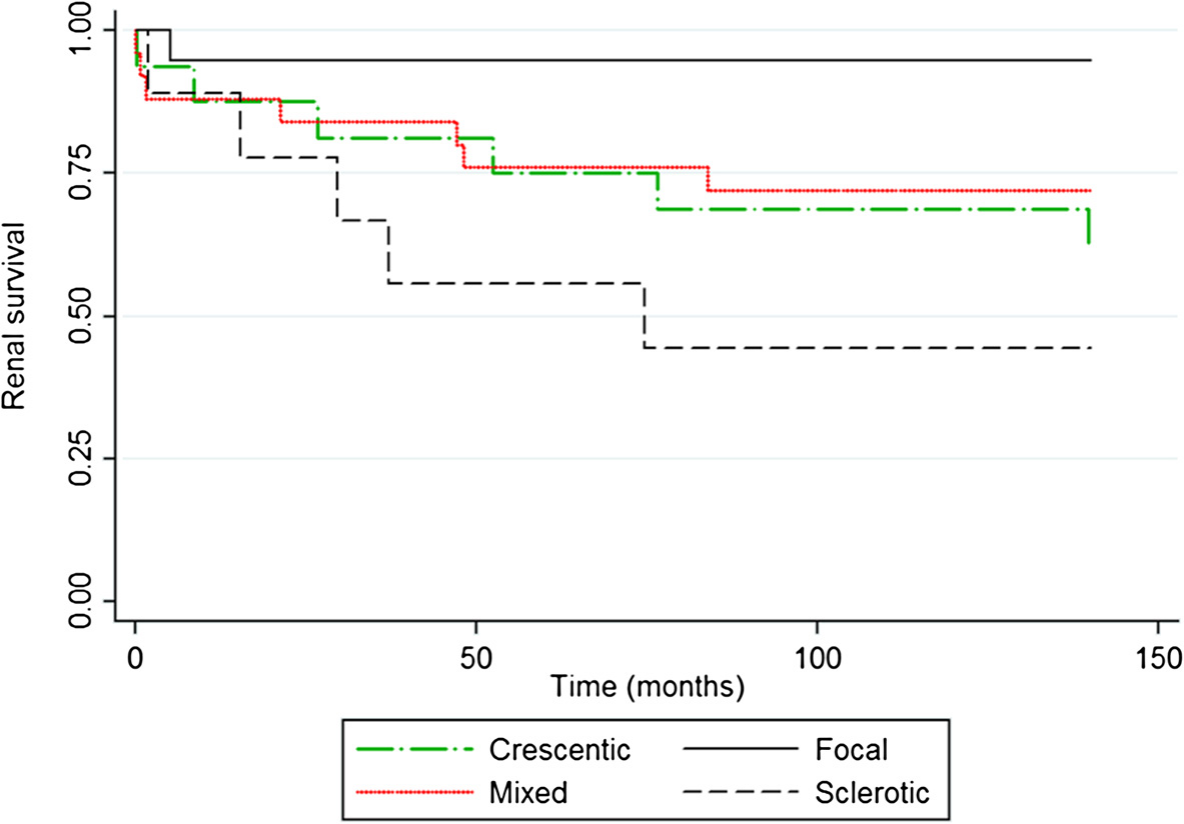
Renal survival is depicted by Kaplan Meier survival estimates as time (in months) to the development of ESRD (defined by need for renal replacement therapy by dialysis or transplant).

Mean e-GFR at 6 months, 12 months, and 24 months is shown in Table 2. The mean change in e-GFR from entry to one year was higher in the focal and crescentic class compared to mixed and sclerotic class (p = 0.37 for the classes). There were 16 patients that reached end stage renal disease at 12 months (dialysis: 15 transplant: 1). Renal survival at one year was 90%, 78%, 81% and 73% for the focal, crescentic, mixed and sclerotic group respectively (p = 0.55) with patients in the focal class having best renal survival and patients in sclerotic class having worse renal survival. Time to development of ESRD stratified by pathologic class is presented in Figure 1. There were 6 deaths with 2 of them occurring within the first year of diagnosis. The cause of death was cardiac arrest in one, aspiration pneumonia in one patient, complications of renal failure in one patient and unknown in the remaining 3 patients.

Age, e-GFR at time of biopsy, and histopathologic classification were assessed as potential predictors of 12 month e-GFR using multivariate linear regression. The results are shown in Table 3. Age and baseline e-GFR were independent predictors of e-GFR at one year. Compared to those with focal GN, patients with crescentic, mixed, and sclerotic biopsies had worse e-GFR at one year post-biopsy.

**Table 3:** Linear regression for outcome eGFR at twelve months among those who did not reach the endpoint of ESRD by twelve months using focal as the reference group

	**eGFR at 12 months (n = 57)**	
	**β**	**p-value**
eGFR at biopsy	0.35	<0.001
Age at biopsy (years)	-0.52	0.003
Crescentic class	-11.35	0.092
Mixed class	-21.25	0.001
Sclerotic class	-23.67	0.006

## Discussion

Pauci-immune glomerulonephritis is a severe manifestation of AAV that is associated with substantial morbidity and mortality. A new histopathologic classification of ANCA-associated GN was proposed for prognostication based on histologic class at diagnosis and validated in 100 patients from two European clinical trials. The present study was designed to validate this classification in a U.S cohort of 76 patients from a single center. The study demonstrates that the e-GFR at diagnosis is highest in patients with focal class and lowest in patients with crescentic class. Age, baseline e-GFR, and histologic class were predictors of e-GFR at one year. Patients with crescentic, mixed, and sclerotic biopsies had worse e-GFR at one year post-biopsy compared to those with focal GN.

The study cohort had a higher proportion of biopsies in the mixed class compared to the initial validation study from EUVAS and the recent observation on the Chinese cohort [22] where there was a predominance of the crescentic class. The proportion of normal glomeruli in the mixed class in our cohort was similar to the Chinese cohort, but higher than the validation cohort from EUVAS. In this U.S, cohort, the baseline e-GFR was highest in the focal class, which had more than 50% normal glomeruli. This is in agreement with the observations in the initial validation study from EUVAS and the recent observation in the Chinese cohort [22]. Previous studies have also correlated baseline e-GFR with the percentage of normal glomeruli [11].

In this U.S. cohort, the crescentic class had the lowest baseline e-GFR. This was similar to the findings in a recently published study that included patients enrolled in the RITUXIVAS trial [23]. There was a predominance of PR3cANCA positivity among those in the crescentic class; a positive correlation between serum creatinine and active glomerular lesions has been shown in previous studies in PR3c ANCA positive patients [10,24]. In contrast, in the Chinese cohort the sclerotic class had the lowest baseline e-GFR. This may reflect a higher representation of MPO-pANCA in the Chinese cohort, as MPO-pANCA patients have been shown in earlier studies to present with more chronic lesions at the time of presentation compared to PR3-cANCA positive patients [25].

Among our 24 patients who were dialysis dependent at presentation, 22 were treated with immunosuppressive drugs and 11 of these patients (50%) recovered adequate renal function to discontinue dialysis. These rates of renal recovery are similar to those reported in some cohorts [9,26,27] though lower compared to other series [28]. Among the dialysis dependent patients, more patients in the focal class recovered renal function compared to patients in the crescentic and mixed class. None of the patients in the sclerotic group recovered renal function.

In this U.S. cohort, the renal biopsy class correlated with e-GFR at one year, and the sequence of class (focal, crescentic, mixed and sclerotic) corresponded to the order of severity of renal dysfunction. These findings are in agreement with the validation study described by Berden et al. [19] but differ from the Chinese cohort where the crescentic group had worse one year renal outcomes compared to the mixed class. A recent analysis of outcomes of ANCA associated glomerulonephritis over the last 3 decades also showed worse renal outcome in the crescentic group compared to the mixed group attributed to the finding of a lower percentage of normal glomeruli in the crescentic group when compared to the mixed group [29].

In linear regression analyses age, baseline e-GFR, and renal biopsy class were independent predictors of e-GFR at one year. The influence of baseline e-GFR on one year e-GFR has been shown in other studies [15,23,25]. In addition, recent analysis of the RITUXIVAS trial patients shows correlation of one year e-GFR with sclerotic glomeruli, baseline e-GFR and age [23]. Renal survival at one year in our cohort was best in the focal class similar to the findings from the Chinese cohort and the initial validation cohort from EUVAS. In this U.S. cohort, patients in the mixed class had similar one year survival compared to crescentic class. This was also demonstrated in the Chinese cohort [22] and the recent series published from the Limburg Renal Registry [30]. In contrast, the crescentic class had better survival when compared to mixed class in the study from EUVAS [19]. This is likely due to a higher proportion of normal glomeruli present in those that were categorized as mixed class in our cohort. The correlation of one year e-GFR and normal glomeruli has been shown in previous studies [10,11,15].

The current cohort study has limitations because of the retrospective nature of the study. Like others, the study is limited by the small patient numbers, making multivariate analysis problematic. Although the study cohort was heterogeneous in terms of initial treatment, all patients were included to represent the spectrum of patients we encounter in routine clinical practice. Despite these limitations, the study provides new data on validation of the proposed IWGRP AAV GN classification in a U.S cohort of patients treated in clinical practice outside of clinical trials.

## Conclusions

In conclusion, we have re-evaluated the new histopathologic classification of AAV GN in a single center cohort in U.S. and there was a demonstrable association between histologic class and renal function at baseline and one year. Patients in the sclerotic class who were dialysis dependent on presentation had no significant response to therapy.

## Competing interest

Duvuru Geetha, M.D.: Served as consultant and received honoraria from Genentech for educational purposes.

## Authors’ contributions

CLE: Participated in data collection, in performance of research and writing of the paper. RLM: Participated in data collection and analysis and writing of the paper. JPH: Participated in data collection, in performance of research and writing of the paper. LCR: Participated in performance of research and writing of the paper. DG: Participated in research design, data collection, performance of research and writing of the paper. All authors read and approved the final manuscript.

## Pre-publication history

The pre-publication history for this paper can be accessed here:

http://www.biomedcentral.com/1471-2369/14/210/prepub
